# Contactless processing of SiGe-melts in EML under reduced gravity

**DOI:** 10.1038/s41526-016-0007-3

**Published:** 2016-12-16

**Authors:** Yuansu Luo, Bernd Damaschke, Stephan Schneider, Georg Lohöfer, Nikolay Abrosimov, Matthias Czupalla, Konrad Samwer

**Affiliations:** 1 I. Physikalisches Institut, Georg-August-Universität, Göttingen, D-37077, Germany; 2 Institut für Materialphysik im Weltraum, Deutsches Zentrum für Luft- und Raumfahrt (DLR), Köln, D-51147, Germany; 3 Leibniz Institute for Crystal Growth, Berlin, D-12489, Germany

## Abstract

The processing of semiconductors based on electromagnetic levitation is a challenge, because this kind of materials shows a poor electrical conductivity. Here, we report the results of measurements of the thermophysical properties obtained recently from highly doped semiconductors Si_1−*x*_Ge_*x*_ under microgravity conditions in the framework of parabola flight campaigns. Due to the limited time of about 20 s of microgravity especially Ge-rich samples with low melting temperatures were investigated. The measurements were performed contactlessly by video techniques with subsequent digital image processing. Linear and volume thermal expansion coefficients were measured hereby from image data. An anomaly of volume changes near the solidus temperature is visible. Viscosity and surface tension were determined by the oscillating drop technique using optic and electronic data. It was observed that the alloying of Si into Ge increases the surface tension of the melts. The viscosity is following an Arrhenius equation and shows a crossover temperature which separates simple liquid at high temperatures from cooperative liquid at low temperatures.

## Electronic materials: Achieving a better mix

Low-gravity environments help to produce a semiconducting alloy with great promise for electronics, shows researchers from Germany. Yuansu Luo from the Georg-August-Universität and co-workers measure the thermal properties of molten silicon–germanium during parabolic flights. Silicon is the dominant material in the electronic industry. Adding germanium, however, creates a semiconductor with even more useful properties. Producing high-quality crystals of this alloy is challenging because gravity separates the two elements when in liquid form. A low-gravity environment could help, but more must be known about the properties of silicon–germanium under such conditions. Luo et al*.* processed a silicon–germanium melt in an electromagnetic levitation facility in microgravity conditions, analyzed video images to determine its thermal expansion, viscosity, and surface tension and observed an alloying effect and a crossover phenomenon. The results pave the way for more detailed investigations on the International Space Station.

## Introduction

The semiconductors Si and Ge are widely used nowadays in almost all modern electronic devices. Their alloys Si_1−*x*_Ge_*x*_ additionally exhibit tunable band gaps and thus are more flexible in applications than the pure silicon technique.^1, 2^ A huge enhancement of charge carrier mobility was additionally observed in Si_1−*x*_Ge_*x*_ in strained heterostructures designed for advanced MOSFET devices.^3, 4^ Near the concentration of Si_50_Ge_50_, the strain effect is maximal. However, due to strong segregation of components in the Si–Ge system, the production of alloy single crystals is not easy. By conventional Czochralski technique^5^ single crystals of alloys could be grown, but only for the case of Ge or Si rich alloys. By traveling liquidus zone method^6, 7^ single crystals of the Si_50_Ge_50_ alloy could be prepared, but the size was limited to 2 mm in diameter due to gravity-driven convection and subsequent segregation. However, the growth of large size Si_50_Ge_50_ single crystals was reported recently on board the ISS under microgravity (µg) conditions.^8^ For the understanding and simulation of the crystal growth the thermophysical properties of Si_1−*x*_Ge_*x*_ melts under microgravity conditions will be increasingly interesting not only for semiconductor industry, but also for fundamental research of material sciences in space environments.

As a preparatory work for planned experiments on board the ISS, this article reports contactless processing of doped Si_1−*x*_Ge_*x*_ melts in an electromagnetic levitation (EML) facility under microgravity conditions in the framework of parabola flights.^9^ Low positioning forces compared to the surface tension of the liquid sample, which keep the levitated drops well spherical, and the absence of gravity-driven convection allow precision measurements of thermophysical properties of Si_1−*x*_Ge_*x*_ melts. Additionally, a deep undercooling of the melt is expected in containerless experiments, giving access to study anomalies like volume changes expected for a liquid–liquid phase transition as theoretically predicted by Angell *et al*.^10^

## Results and discussion

### Processing

The processing of a sample in EML during the about 20 s lasting low gravity phase of one parabola fight consists essentially of three steps (cf. also Fig. 3):

1. Contactless positioning of the sample in the center of the levitation coil by the quadrupole shaped high frequency magnetic “positioning field”.
2. Inductive heating of the sample beyond the melting temperature by the additional dipole shaped high frequency magnetic “heating field”.
3. Deactivation of the heating field and reduction of the positioning field, so that the liquid sample cools freely down. During this phase of minimal external impact the different experiments at the sample are performed.

**Fig. 1:**
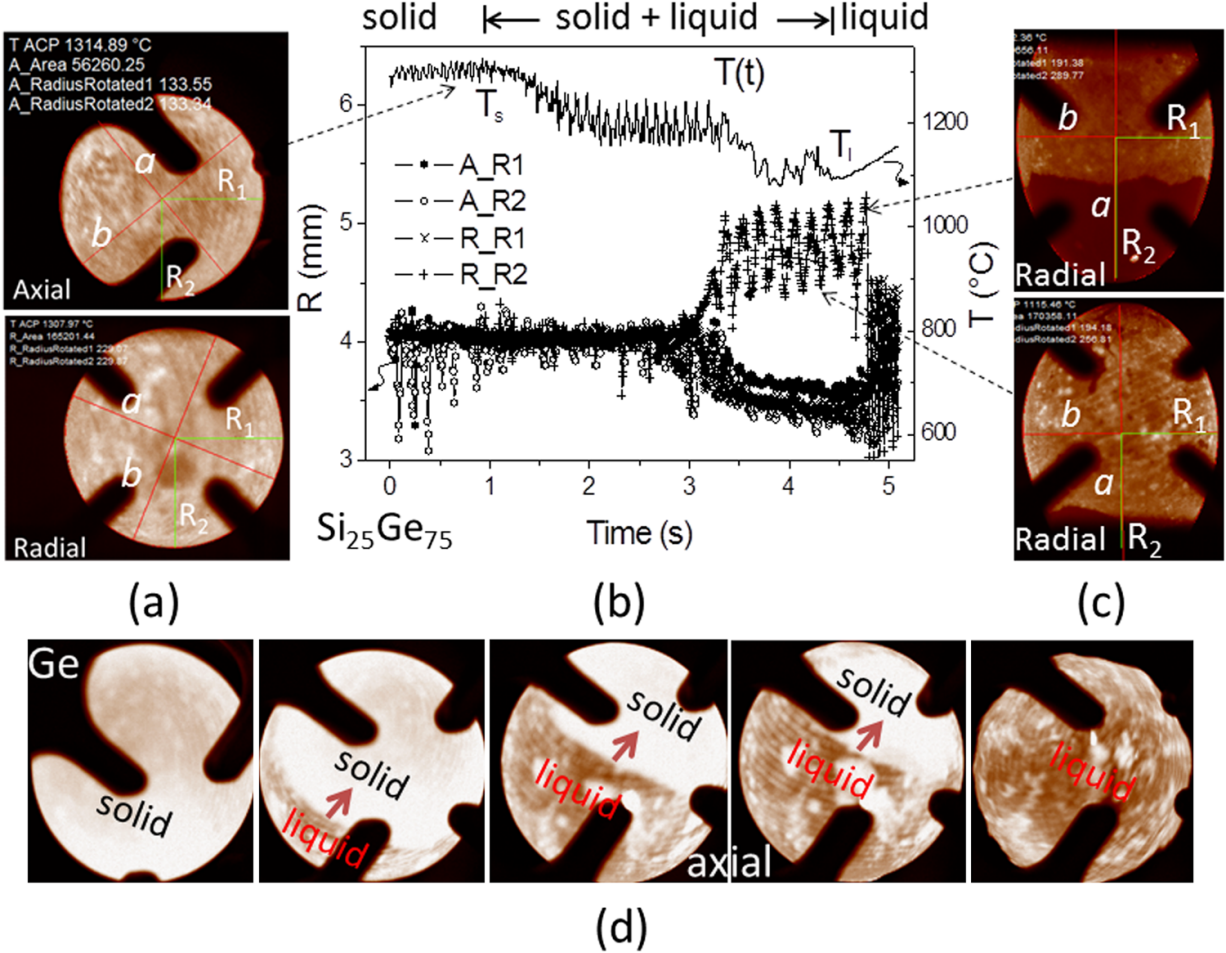
**a** Axial and radial images of Si_25_Ge_75_ near the solidus temperature *T*_s_. **b** Profiles of temperatures and radii measured upon heating. **c** Radial images of the ellipsoidally deformed molten sample near the liquidus temperature *T*_*l*_. **d** Axial images of doped Ge during melting with *bright* and *dark* domains due to solid and liquid phases, the arrow indicates the rotation direction

Figure 1 demonstrate behaviors of the 1st processing cycle of doped Ge and Si_25_Ge_75._ The traces on the sample surface caused by machining are visible. Images of the spherical sample were recorded upon heating near the solidus temperature *T*_s_, where the solid semiconductors could be positioned in EML due to enhanced *σ* at high temperatures. In the two-phase state, the images show bright and dark domains corresponding to the solid and liquid phases, respectively, reflecting the difference in spectral emissivity *ε* (for instance, *ε* = 0.39 measured for solid Ge and 0.21 for liquid Ge).

By means of a digital procedure with the software “TeVi” (of SEA Datentechnik GmbH), the edge of the sample was fitted with a curve *R*(*θ*)*,* which is a polynomial function in polar coordinates with the origin located in the center of the sample.^11^ Figure 1a shows the fitted edge curves (red circles) for the axial and radial images obtained from doped Si_25_Ge_75_ near *T*_s_. The fitting yields half axes *a*, *b* (red), horizontal radius *R*_1_ and vertical radius *R*_2_ (green) of the sphere, including areas (A) which give an effective radius *r*_eff_(A = π$\,{r}_{{\rm{eff}}}^{2}$). For a sphere, of course all length data *a*, *b*, *R*_1_ and *R*_2_ are identical. Figure 1c illustrates radial images of the molten sample close to *T*_l_ coated with a small fraction of a solid phase (mainly surface oxide). The sphere became ellipsoidal due to the uniaxial deformation by the dipole heating field. Note, the radial images shown in Fig. 1c are incomplete due to a shadowing by coil windings leading to problems in the relevant radii evaluated.

Figure 1b demonstrates the time-temperature profile *T*(t) measured for doped Si_25_Ge_75_. Upon heating the temperature rises in the period 0 < *t* < 1s and reaches a plateau corresponding to the solidus point *T*_s_, where the sample starts to melt. The profile near *T*_s_ is less fluctuated, indicating the stable positioning of the solid semiconductor in EML. Because of a lower emissivity of the liquid phase, the profile *T*(t) apparently decreases during the melting and reaches subsequently a minimum corresponding to the liquidus point *T*_l_. Because a fixed spectral emissivity of 0.20 (*ε*_P_) was used for this cycle, the displayed minimum temperature of about 1100 °C (*T*_lP_) is close to the liquidus temperature *T*_l_ = 1115 °C expected for the sample Si_25_Ge_75_ (see Table 1). The correct *ε* can be estimated hereby from Stefan Boltzmann law $\varepsilon ={\varepsilon }_{{\rm{P}}}{{T}_{{\rm{lP}}}}^{4}/{T}_{{\rm{l}}}^{4}$ and has a value of about 0.19. As mentioned below, the molten sample is metallic and expectedly shows a high electromagnetic coupling to the heater coil. As a consequence, the relevant *T*(t) profile rises quickly and smooth. Note, the fluctuation between *T*_s_ and *T*_l_ is due to the rotation of the two-phase sample consisting of bright and dark domains (Fig. 1d) regarding the different spectral emissivity mentioned above. The period determines the rotation rate. It is about 9.1 Hz. The rotation rapidly slows down to 0.5–1.0 Hz as soon as the sample is molten.

**Table 1:** Solidus and liquidus temperatures *T*_s_/*T*_l_, dopant concentration and electrical conductivity *σ* as well as energy gaps *E*_g_ of Si_1−*x*_Ge_*x*_ semiconductor samples used

Samples	Real *x*, at.% Ge	*T*_s_/*T*_l_ (°C) for real *x*	Density *ρ* (g/cm^3^) at RT/*T*_l_	Doping (at./cm^3^)	*σ* (Ω^−1^cm^−1^) at RT	Energy gap *E*_g_ (eV) at RT/*T*_l_
Ge	–	937	5.34/5.57	Sb, *n* = 1.0 × 10^19^	1.0 × 10^3^	0.66/0.26
Si_10_Ge_90_	89	963/1028	5.06	B, *p* = 1.9 × 10^20^	1.7 × 10^3^	0.71/0.29
Si_25_Ge_75_	77	1000/1116	4.73/5.07	B, *p* = 1.5 × 10^20^	1.3 × 10^3^	0.79/0.34
Si_50_Ge_50_	48	1118/1283	3.95/–	B, *p* = 1.9 × 10^20^	1.7 × 10^3^	0.91/0.43
Si_75_Ge_25_	26	1238/1364	3.15/–	B, *p* = 1.65 × 10^20^	1.4 × 10^3^	1.02/0.51

### Density and thermal expansion

The elements Si and Ge show—similar to water—a density anomaly near the transition temperatures. The phenomenon is already reflected in densities of the samples given in Table 1 and is actually visible in Fig. 1b: Despite the increasing temperature at the beginning of the measurement, the value for the radius of the sphere decreases from slightly above 4 mm down to slightly below when it approaches *T*_s_. Using the data given in Fig. 1b, the volumes of the solid and liquid sample can be estimated to be about 0.274 cm^3^ at *T*_s_ and 0.252 cm^3^ at *T*_l_, which is an abnormal volume change caused by solid–liquid phase transition of about 8.0%. The result suggests a local minimum of the density of the solid material near *T*_s_ and a corresponding local maximum of the density of the liquid near *T*_l_.

**Fig. 2:**
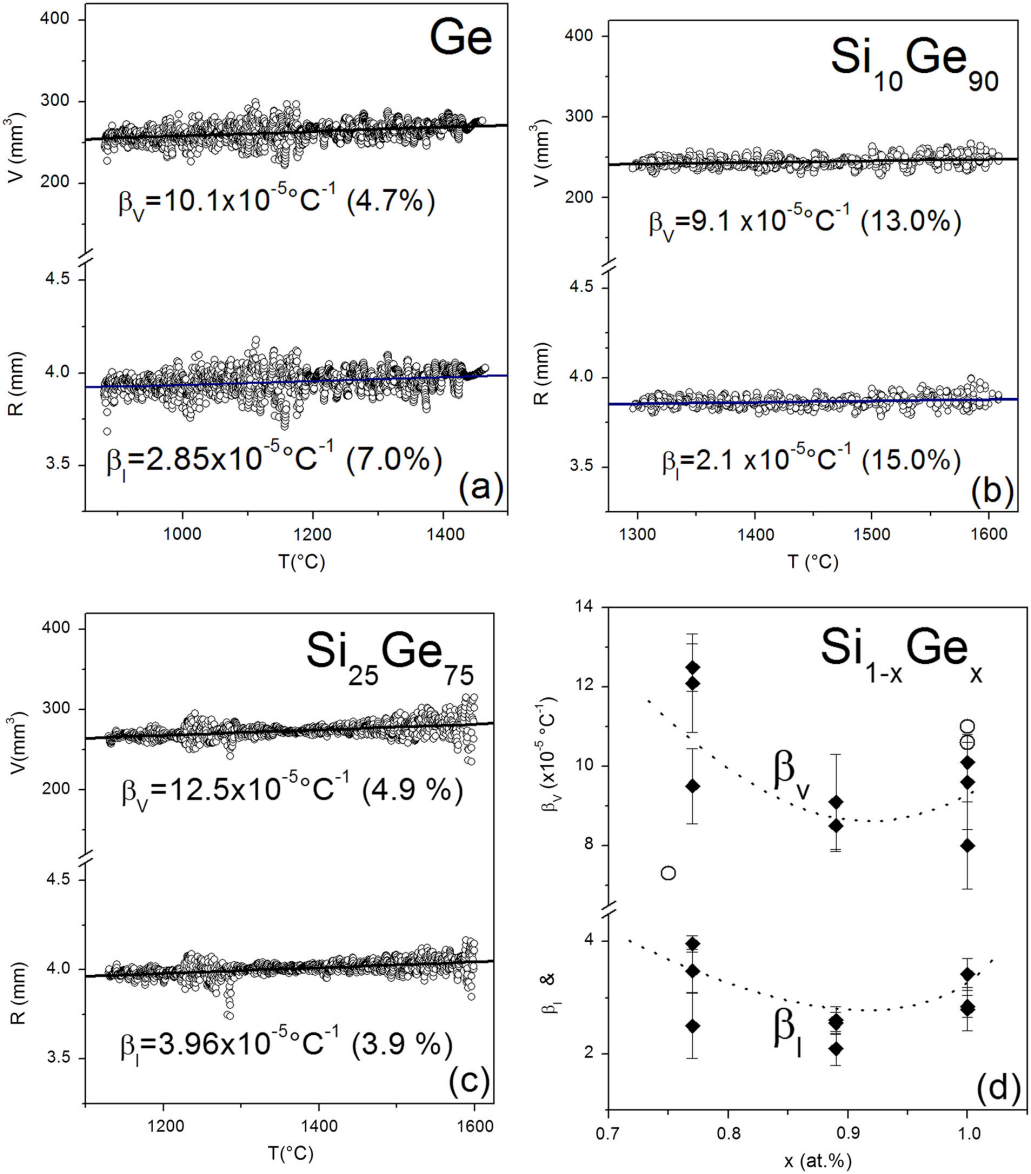
Mean radii and volumes for Ge (**a**), Si_10_Ge_90_ (**b**), and Si_25_Ge_75_ (**c**) as a function of the temperature. The thermal expansion coefficients *β*_*v*_ and *β*_*l*_ (**d**) were determined by linear fits. The three additional data (*circles*) stem from refs 12,13

The evaluation of the thermal expansion is disturbed by sample movements and rotations due to µg-disturbances and the limited time of about 20 s for a melt cycle. In Fig. 2 data of the radii measured for pure Ge, Si_10_Ge_90_ and Si_25_Ge_75_ are presented. They show an almost linear temperature development over the whole measured temperature range, and therefore the expansion coefficients are quasi temperature independent for the existing measurement accuracy. The dependence of the thermal expansion coefficients on the Ge-concentration is shown in Fig. 2d. For a definitive discussion and interpretation the number and accuracy of the data points have to be improved. Precision experiments on board the ISS are planned.

The Ge-rich samples are well suited for the parabola flight experiments due to their relatively low melting temperatures, which can be reached fast. Therefore, there is enough time for the cooling process available.

### Viscosity and surface tension

**Fig. 3:**
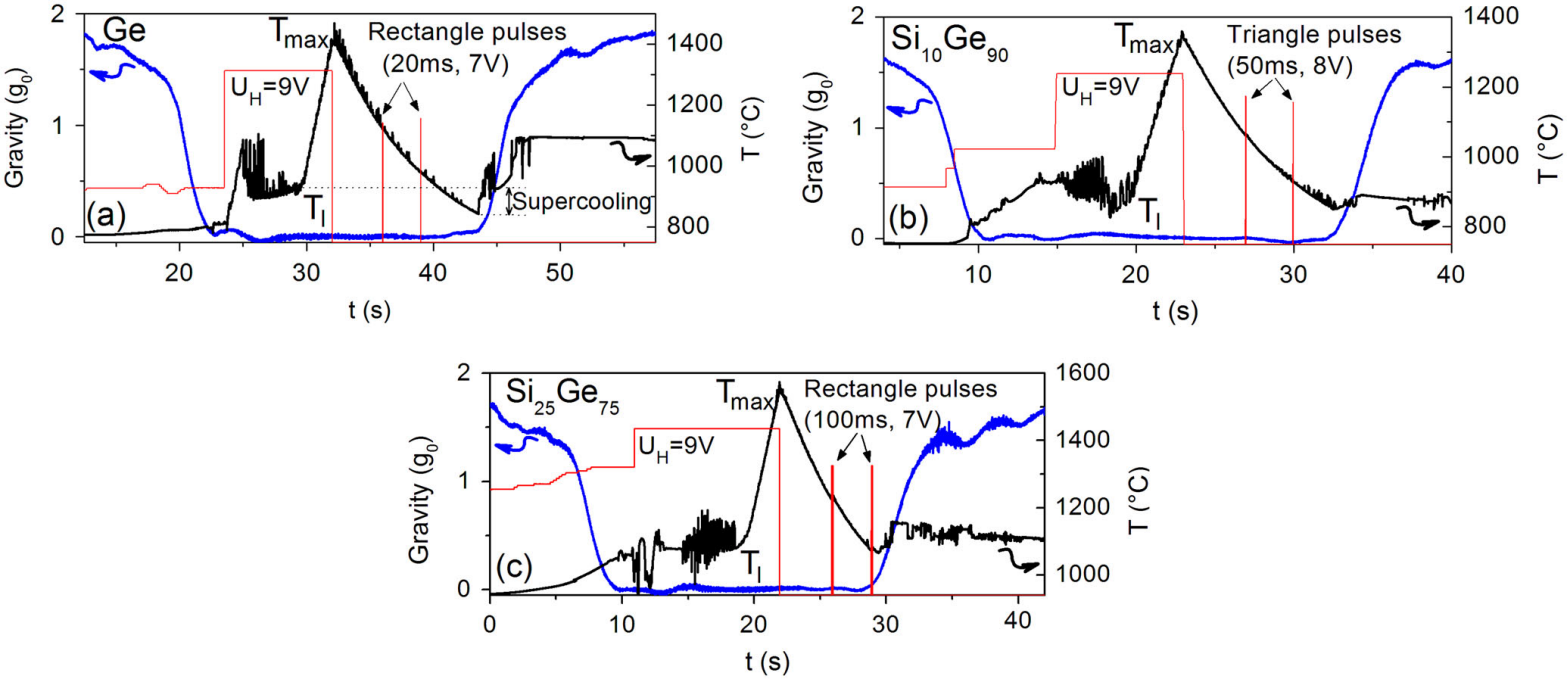
Time profiles of the µg-levels (*blue*), the heating voltages (*red*) as a measure of the magnetic field strength and the temperatures (*black*) measured for doped Ge (**a**), Si_10_Ge_90_ (**b**), and Si_25_Ge_75_ (**c**). For the excitation of drop oscillations various pulse shapes (*red*) were used.

The viscosity and the surface tension were measured with the oscillating drop technique. For this purpose the melted drop was squeezed by a short pulse of the magnetic heating field which resulted in surface oscillations during its relaxation to the equilibrium spherical shape. From the decay of the amplitude of these oscillations the viscosity and from the resonance frequency the surface tension can be derived.^14^ For the optimization of the excitation various pulse shape and heights were tried as indicated in Fig. 3.

In Fig. 3 the melting plateaus with the accompanying jump in the emissivity and the connected disturbances in the pyrometer signal can be again observed. The *g*-level in the measuring phase is less than 0.05 g. After switching off the heater the sample was excited to oscillations which can be used for the evaluation of the viscosity *η* and surface tension *γ*. Additionally, heater pulses with various height and shape were applied at different temperatures. All pulses were able to excite oscillations; for long pulses a small temperature increase was observed.

**Fig. 4:**
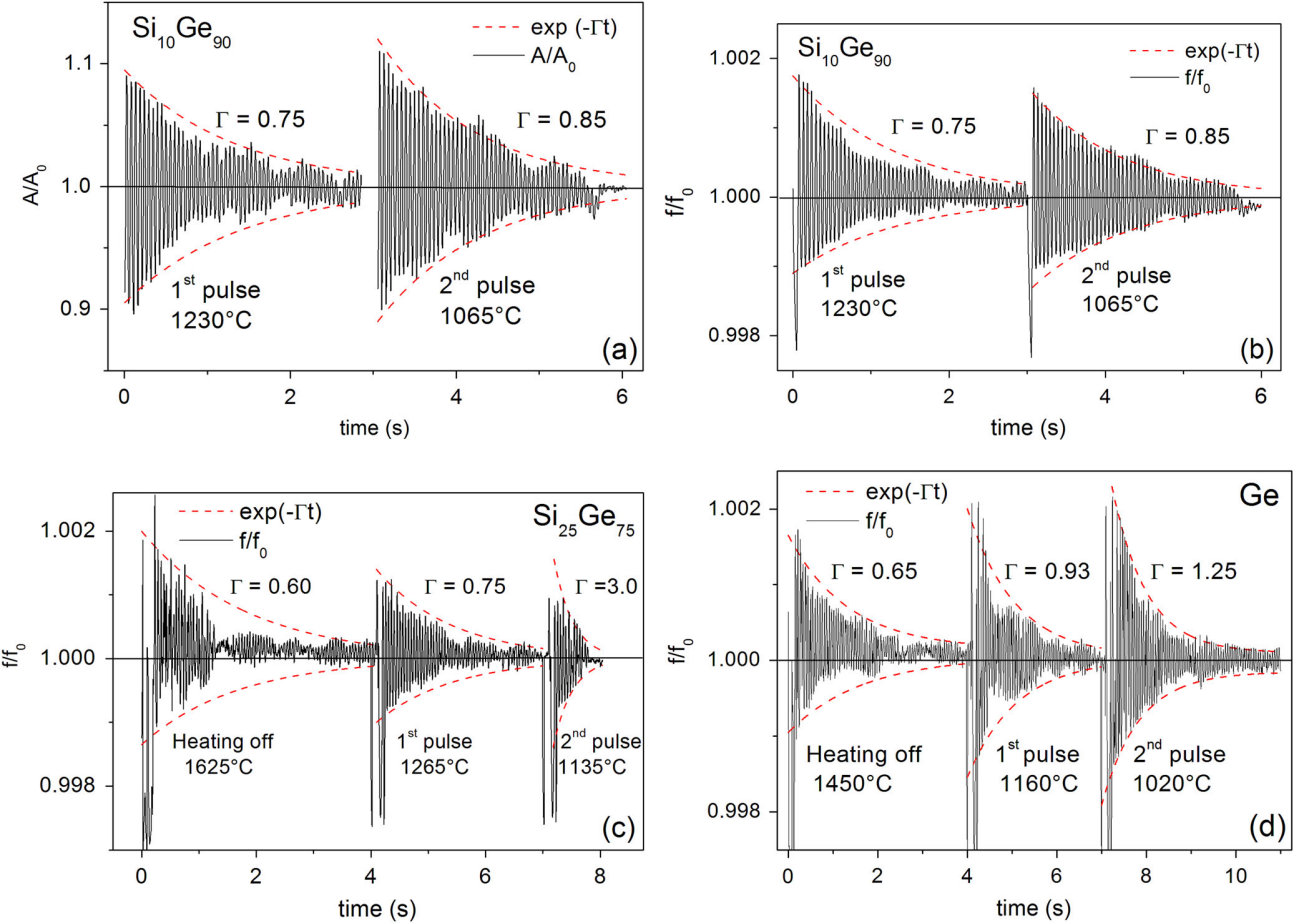
Oscillations of optic (**a**) and electronic (**b**) data measured for the Si_10_Ge_90_ drop with triangular pules (8 V, 50 ms), as well as electronic data for the Si_25_Ge_75_ (**c**), and Ge (**d**) drops with rectangle pulses (7 V, 100 ms), including fittings (*red dashed lines*) with an exponential decay.

Figure 4 illustrates typical surface oscillations of levitated Ge, Si_10_Ge_90_ and Si_25_Ge_75_ drops, excited by heating off and extra pulses in the heater coil at different temperatures. The resonance frequency *ν* and the damping *Γ* are functions of the surface tension *γ* = (3π*M*/8)*v* ^2^ and the viscosity $\eta =(3M/20\,\pi {r}_{0})\Gamma$ *,* respectively, where *M* denotes the mass and *r*_0_ the radius of the drop. The usage of these simple relations assumes that the drop is spherical and force free under microgravity. The linear relation of *η* to *Γ* is valid only when *η* < *η*_crit_, where the threshold *η*_crit_ = 0.76(*γρr*_0_)^1/2^ ^14^and it approximately equals to 2.9 Pa s, as estimated from the measured data *γ* ≈ 0.7 Nm^−1^, *r*_0_ ≈ 4.0 mm, and *ρ* ≈ 5.2 g/cm^3^. As will be shown below, the *η* value measured for relevant samples is less than 0.1 Pa s and far below *η*_crit_.

In Fig. 4 typical oscillations and their decay are presented as a function of time. In Fig. 4a the data *A*/*A*_0_ were taken from optical measurements of the axial camera. They are the cross section of the Si_10_Ge_90_ sample perpendicular to the heating field and normalized to the mean value. In the EML facility for the parabola flights the data can also be taken electronically with the help of a designed sample coupling electronics (SCE) (Fig.e 4b–d), leading to a similar behavior. The data were fitted with an exponential decay to extract the viscosity. The corresponding simulations are drawn in Fig. 4 with red dashed lines. A drawback arises from translational and rotational movements of the sample leading to artefacts in the oscillations (Fig. 4c,d) and complicates the evaluation.

**Fig. 5:**
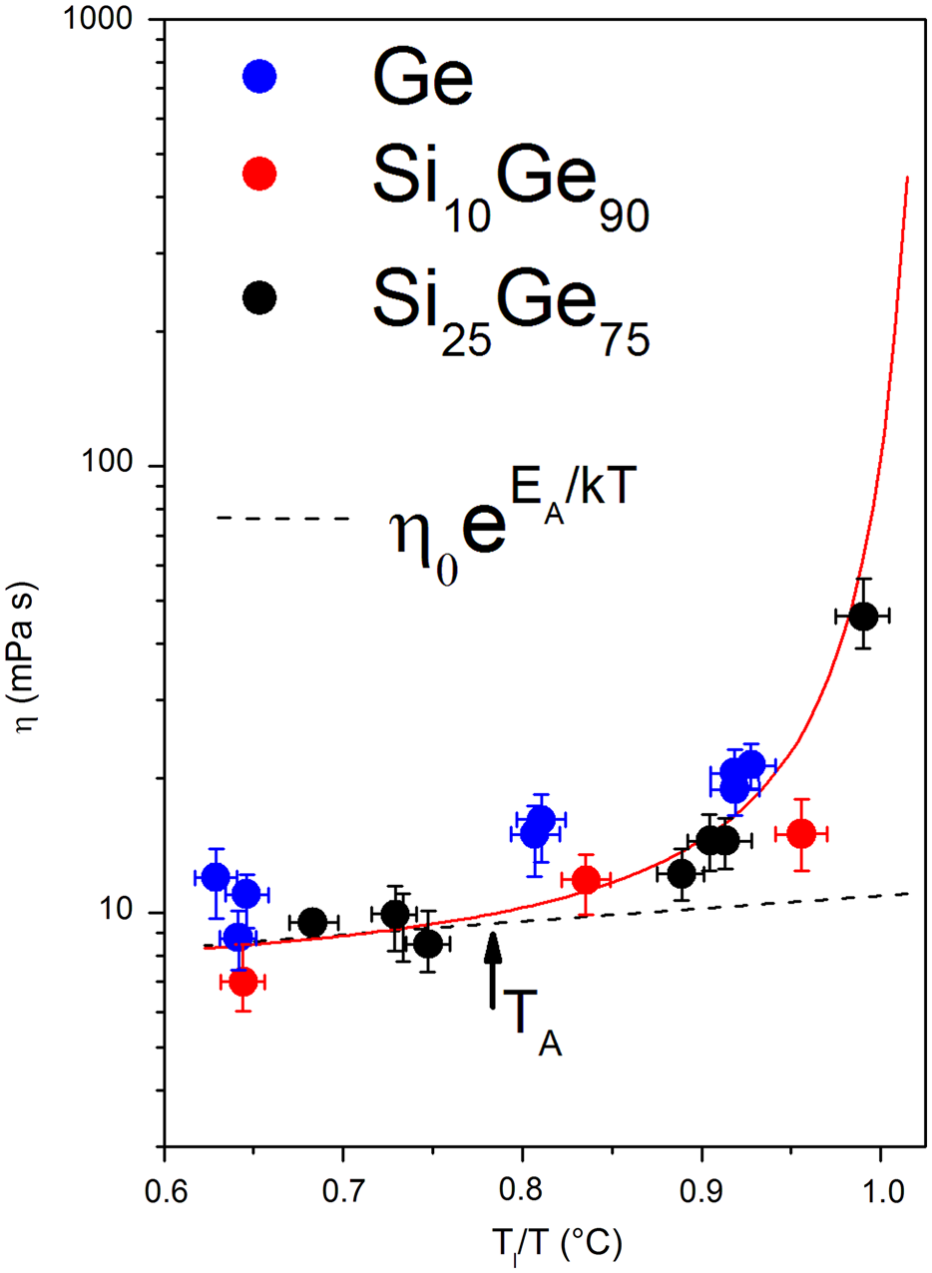
Viscosities *η* measured at different temperatures for Ge, Si_10_Ge_90_, and Si_25_Ge_75_ melts, showing Arrhenius-type behavior (*dashed lines*) with a crossover temperature *T*_A_ between simple and cooperative liquid.^15^

Figure 5 shows as a summary the viscosity data as a function of temperature. With the available measurement accuracy, the viscosity values can be fitted by an Arrhenius function, *η* = *η*_0_ exp(*E*_A_/kT), with activation energy *E*_A_ of about 90–100 meV at high temperatures (left side). Near *T*_A_ the begin of a strong increase is indicated (right side). More data are needed to support this trend, but the behavior may be interpreted in terms of a crossover temperatur *T*_A_ separating a simple fluid from a cooperative behavior known from glass forming melts.^15^ Of course the data especially near *T*_l_ are not sufficient for the interpretation but additional experiments will be performed on board the ISS. For technological applications the knowledge of the viscosity is indispensable. The value of the high temperature limit *η*_0_ was found to be about 5 mPa s, somewhat higher than that (~1 mPa s) measured in vacuum by the electrostatic levitator (ESL) facility using undoped samples.^16^

**Fig. 6:**
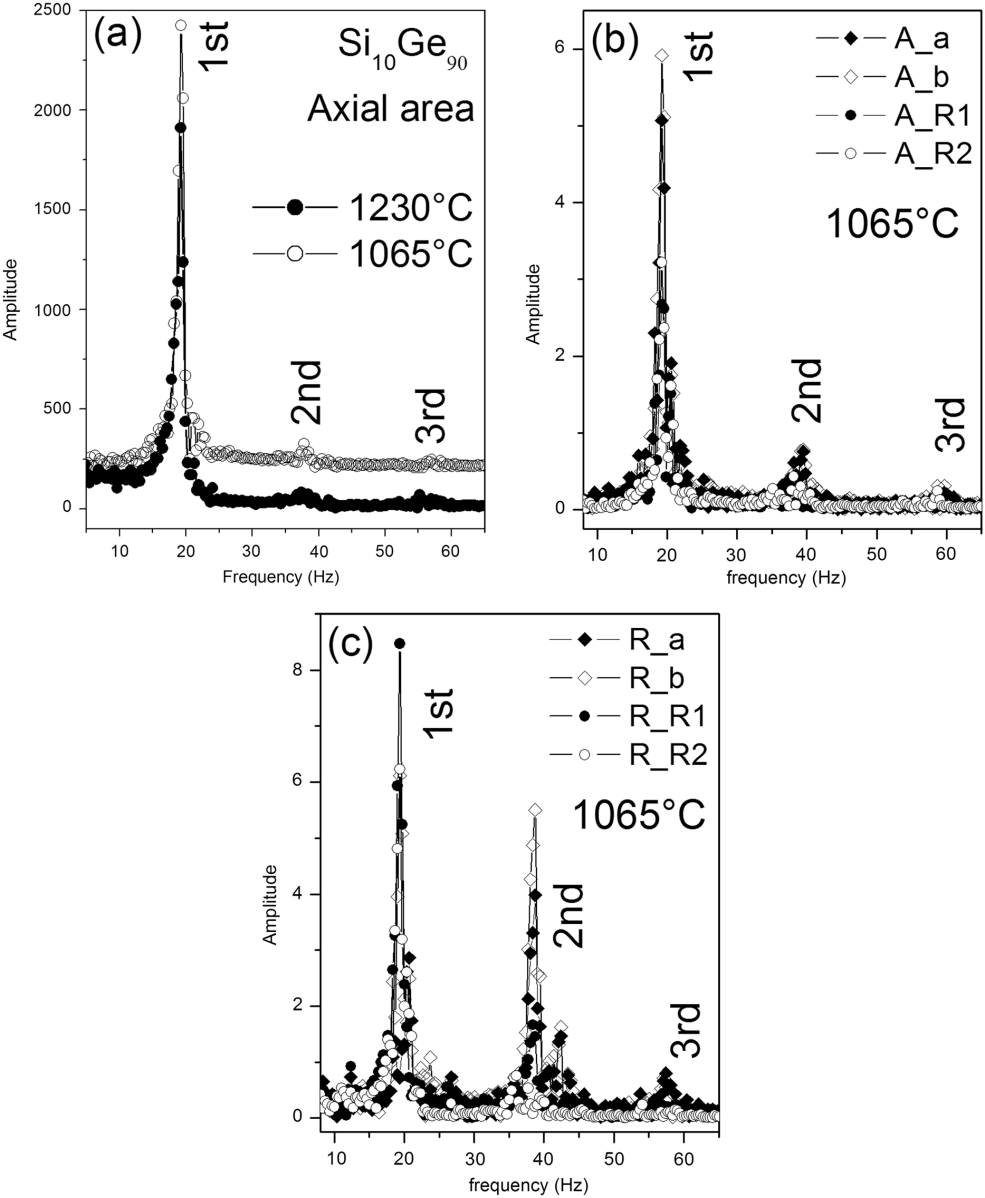
Frequency spectra of the surface oscillations of the liquid Si_10_Ge_90_ droplet excited by triangular pulses at 1230 °C and 1065 °C. The plots of diagram **a** result from data of the sample cross section obtained by the axial camera. The plots of **b** and **c** from length data a, b, *R*_1_, and *R*_2_ obtained by the axial and as well as the radial camera.

The frequency spectra for the determination of the surface tension were deduced by fast Fourier transform (FFT) of the oscillation data. Figure 6 shows frequency spectra for the Si_10_Ge_90_ sample with the resonance peak at 19 Hz and additional higher harmonic peaks. For the analysis the motion of the sample has to be considered carefully, because also an artificially peak due to sample rotation is seen in Fig. 6c at 42 Hz.

**Fig. 7:**
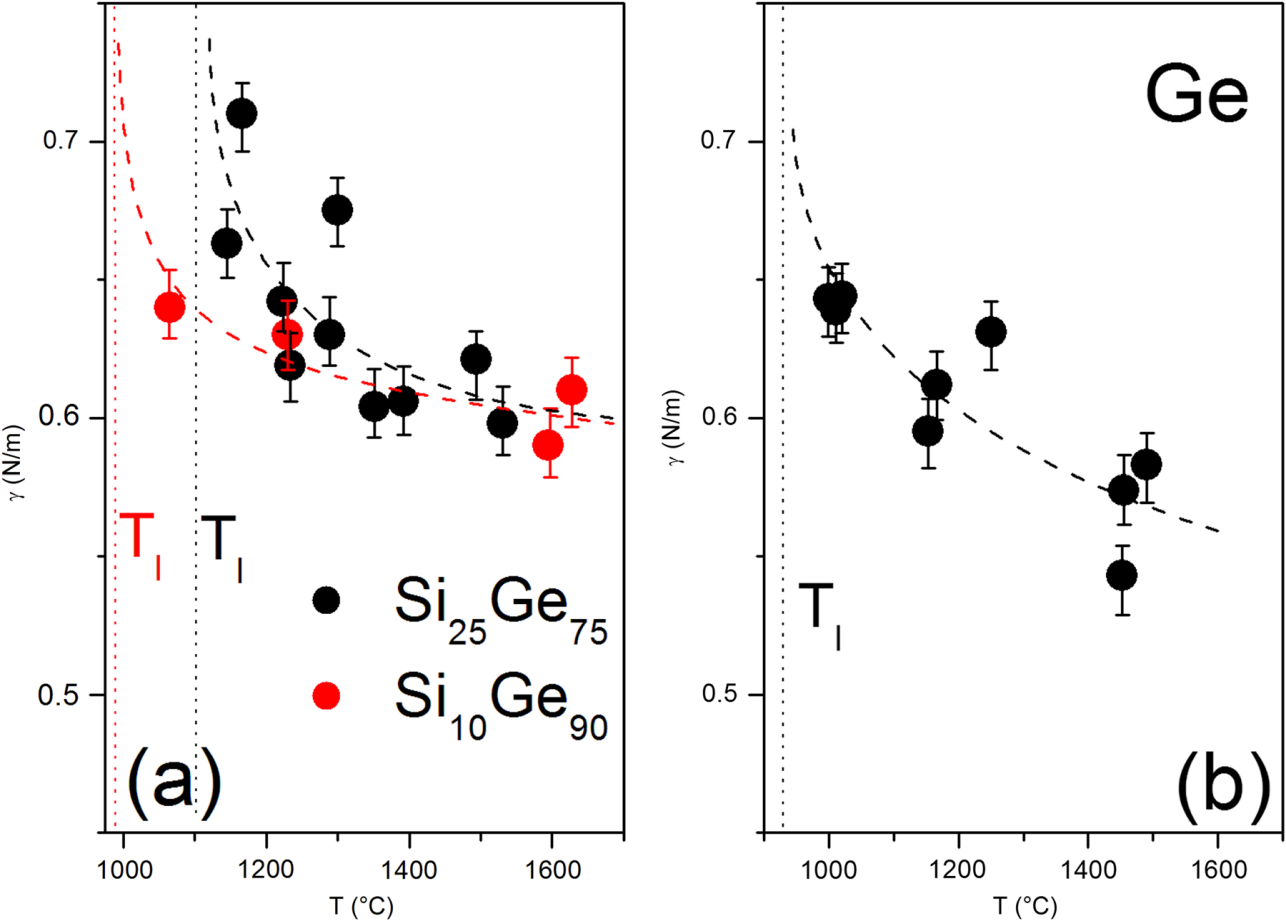
Temperature-dependent surface tension γ measured **a** for Si_10_Ge_90_ and Si_25_Ge_75_ and **b** for the pure Ge (the *dashed lines* only serve as a guide for the eye)

In Fig. 7 the surface tension data are plotted as a function of temperature. As expected, the values strongly increase with decreasing temperature. Additionally, there is evidence for an increase of these values for the alloys compared with the pure Ge. This effect should be investigated in more detail in future measurements on the ISS before a concluding interpretation should be given.

## Experiments

### EML facility and electrical conductivity of Si and Ge

**Fig. 8:**
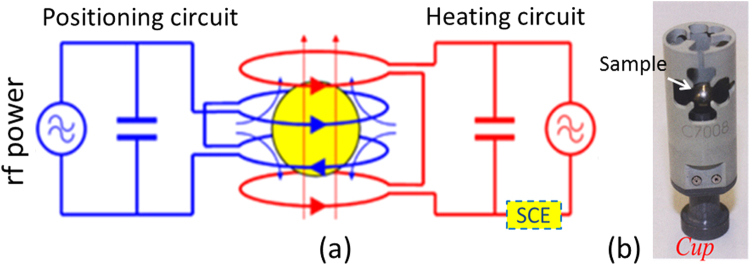
**a** Scheme of the EML facility consisting of a positioner coil (*blue*), the *rf* current through which generates an electromagnetic quadrupole field, and a separated heater coil (*red*), the *rf* current through which generates an electromagnetic dipole field. Included in the heating circuit is the sample coupling electronics (SCE) sensing feedback signals from the sample. **b** Picture of the ceramic cup sample holder enclosing the sample for safety reasons (taken from ref. 17).

As schematically presented in Fig. 8, the EML facility provides a radio frequency (rf) electromagnetic dipole heating field and a rf electromagnetic quadrupole positioning field, which are generated by currents through two appropriate coils connected with two separate power supplies. The heating field circuit is additionally equipped with an electronics (SCE) which is able for sensing changes of resistance and inductance of the sample-coil system and allows an evaluation of the electrical conductivity and the thermal expansion of the sample. The alternating electromagnetic fields induce eddy currents in the sample, heating it up and giving rise to positioning (Lorentz) forces that center the sample in the middle of the coil center where also the focus point of two CCD cameras is located. Clearly, a sufficient electrical conductivity *σ* of the samples is necessary for their processing in the EML and it works thus well for metallic samples (*σ* ≈ 10^4^–10^5^ Ω^−1^cm^−1^). Since pure semiconductors possess a low *σ* only (10^−5^ and 10^−2^ Ω^−1^cm^−1^ for Si and Ge at 300 K, respectively), their processing in EML is a great challenge. The ESL facility^16^ is an alternative for contactless processing of low *σ* materials, but it exclusively works under high vacuum and thus has problems with materials with a high vapor pressure like Si.

Pure Si and Ge are intrinsic (*i*-type) semiconductors and their conductivity rises drastically with temperatures due to thermal excitations of electrons from valence band into conduction band, which simultaneously produce holes in relevant valence band. The density of intrinsic charge carriers *n*_i_ is an exponential function of the temperature *T*. At 300 K, *n*_i_ is about 2 × 10^13^ cm^−3^ for Ge and 0.9 × 10^10^ cm^−3^ for Si, the difference of about three orders of magnitude is reflected in the *σ* values mentioned above. The density *n*_i_ rises rapidly with *T* and reaches up to ~10^19^ cm^−3^ near the melting point *T*_l_ of Ge (937 °C) and Si (1412 °C), which corresponds to a high *σ*. Fortunately, Si and Ge reveal a semiconductor-metal phase transition upon melting due to disordering.^18, 19^ The relevant conductivity jumps up to about 10^4^ Ω^−1^cm^−1^ ^20, 21^and liquid Si and Ge become easy to process in EML. Because of the low *σ* at room temperature, the initial heating of *i*-type semiconductors in the EML facility is difficult. A high-power laser could be used for preheating the samples,^12^ but is not available on board the ISS. Contact with a hot graphite pellet is another way for preheating undoped semiconductors. The hot contact however may lead to contaminations in the reactive samples. Due to these circumstances, we used extrinsic semiconductors, namely highly doped samples. The doping drastically changes the electrical properties of the semiconductors and enables them for processing in the EML.

Parabola flight experiments were performed on board the Zero-g aircraft (Novespace France).^9^ It provides a microgravity phase of about 20 s with a *g*-level of less than 0.05 g, followed with a hypergravity phase (1.8 g). Due to the limited time, low *T*_l_ samples Ge, Si_10_Ge_90_ and Si_25_Ge_75_ are more suitable here than high *T*_l_ samples Si, Si_75_Ge_25_ and Si_50_Ge_50_. The processing was made under Argon gas (350 mbar). It reduces the thickness of deposition layers on the EML coils and increases the cooling rate to ensure the solidification of the melt before the subsequently following hypergravity phase. The surface tension γ and the viscosity *η* of levitated liquids were measured by means of oscillating drop techniques,^14, 22^ using both video and SCE data.

### Sample preparation and characterization

**Fig. 9:**
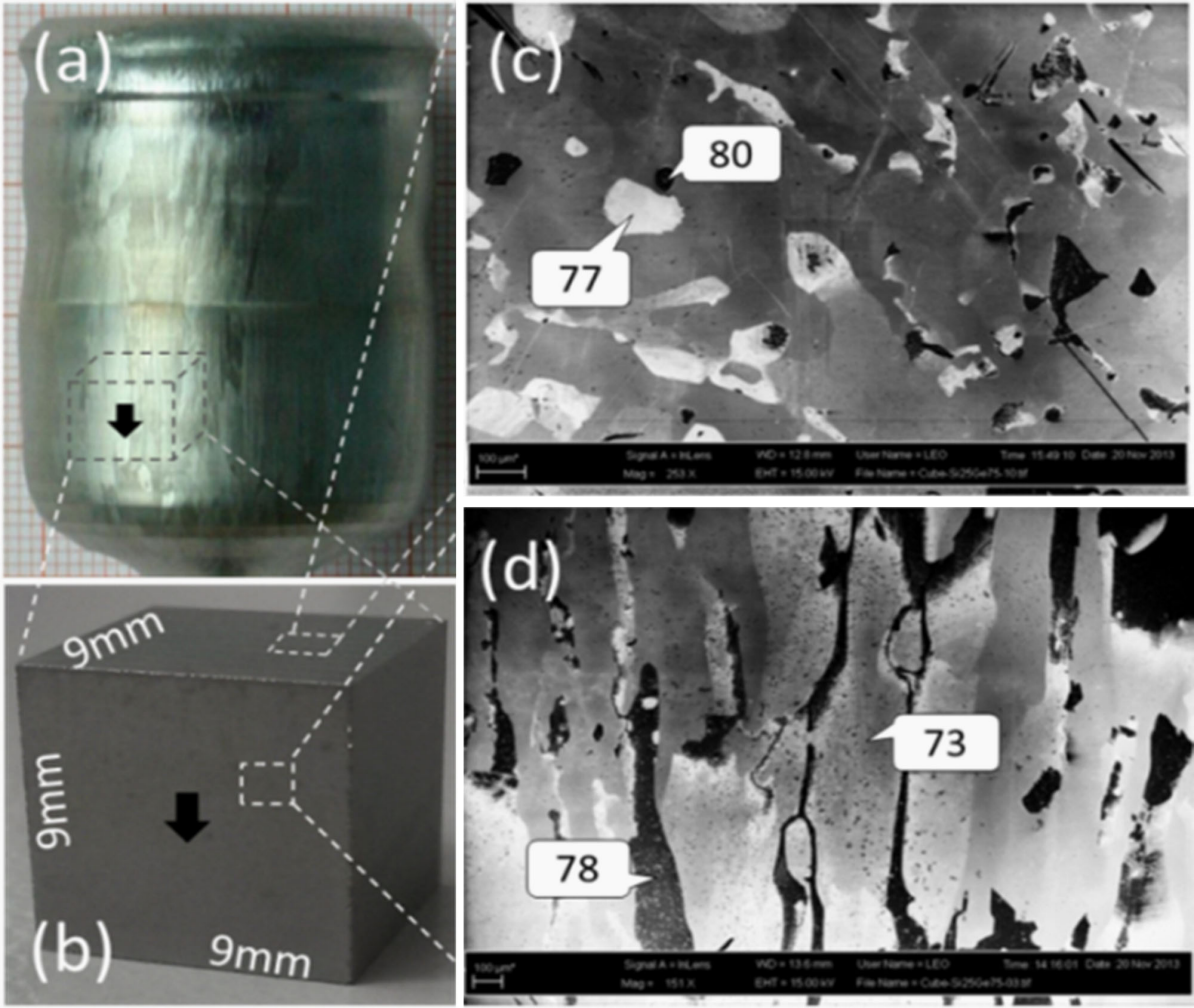
**a** Cylindrical crystal (35 mm in diameter) of a SiGe alloy prepared by Czochralski process, the *arrow* indicates the pulling direction. **b** A cube (9 × 9 × 9 mm³) cut from the cylinder. **c** and **d** SEM images from the *cube top* and *side*, respectively, including EDX data in at.%. The surface of the cube was etched in 30% H_2_O_2_.

Highly doped Ge and Si_1−*x*_Ge_*x*_ alloys were prepared for contactless experiments under microgravity on board the ISS. The alloy crystals were grown by Czochralski technique^5^ with dopant B (acceptor, *p*-type); Ge doped with Sb (donator, *n*-type) is commercially available. Figure 9a shows a cylindric alloy crystal prepared with a pulling rate of about 0.7 mm/h. The low pulling rate is essential to minimize the unavoidable component segregation, which gives rise to constitutional supercooling in front of liquid–solid interface and transfers the crystal growth from single crystalline into polycrystalline. Figure 9c and d present scanning electron microscopy (SEM) images measured for a Si_25_Ge_75_ crystal, showing indeed a polycrystalline structure with growth pillars along the pulling direction. The sample used for SEM images is a cube (9 × 9 × 9 mm^3^) (Fig. 9b) cut from the cylindric crystal. The surface of the cube was treated by mechanical polishing and wet-etching in 30% hydrogen peroxide (H_2_O_2_) solution. The H_2_O_2_ selectively etches Ge^23^ and thus produces the contrast on the surface. The dark (deep) domains correspond to grains containing more Ge, and vice versa. Local EDX-analyses on dark and bright domains were made, showing a difference of the Ge content of 3–5 at.%.

Moreover, a component gradient of 2–5at.% cm^−1^ along the pulling direction occurs. It arises from the segregation of component. Because the segregation coefficient of Ge in Si is less than 1, the end of the cylindrical crystal contains more Ge. The EDX data given in Fig. 9c and d indicate the gradient, the Ge content measured at the cube top surface is slightly higher than that measured at the bottom. Due to the liquidus–solidus distance in the phase diagram, the maximal gradient of 5 at.% cm^−1^ was found in the Si_50_Ge_50_ crystal, while it is only 2–3 at.% cm^−1^ in Si_25_Ge_75_ or in Si_75_Ge_25_. For this reason, the actual concentration of the sample may be deviated by maximal ±2.5 at.% from the nominal concentration given in Table 1. The inhomogeneity in the samples does not influence a multicycle processing in EML. Actually, after a melting cycle the segregation of components in alloys always happens during rapid solidification.

Instead of EDX analysis, which gives a local concentration only, the density data measured for cubic samples were used for calculation of the mean concentrations, i.e. real *x* (see Table 1). The solidus and liquidus temperatures given in Table 1 were calculated accordingly using relations *T*_s_ ≈ *T*_*l*_^Si^ − 738*x *+ 263*x* ^2^ and *T*_l_ ≈ *T*_*l*_^Si^ − 80*x *− 395*x* ^2^, where *T*_l_^Si^ is the melting point of Si (1412 °C).^24^

Samples used for contactless processing in EML are spherical with a diameter of 8 mm. They were machined from cubes (9 × 9 × 9 mm^3^) by ultrasonic milling.

## Conclusion

The melts of highly doped semiconductor samples Ge and Si_1−*x*_Ge_*x*_ were investigated successfully on parabola flights in an electromagnetic levitator. The experiments were performed to prepare upcoming precision experiments on board the ISS and to do first measurements of the thermal properties of the samples in reduced gravity. Due to the limited time for processing in parabola flights only low melting Ge-rich samples were investigated. First data for the density, thermal expansion coefficients, viscosity and surface tension were obtained. In the density data indications for the expected abnormal volume changes were found. A crossover phenomenon separating the simple liquid from the cooperative behavior and an Arrhenius behavior can be deduced from viscosity measurements. The surface tension is strongly temperature dependent and additionally shows an alloying effect. For a conclusive interpretation more precise data are needed and will be measured in next future on board the ISS.
